# Bony destructive injuries of the calcaneus: long-term results of a minimally invasive procedure followed by early functional exercise: a retrospective study

**DOI:** 10.1186/1471-2482-14-19

**Published:** 2014-04-11

**Authors:** Yanling Su, Wei Chen, Qi Zhang, Song Liu, Tao Zhang, Yingze Zhang

**Affiliations:** 1 Department of Orthopaedic Surgery, the Third Hospital of Hebei Medical University, Shijiazhuang, Hebei 050051, P.R. China

**Keywords:** Calcaneal fracture, Bony destructive injury, Internal compression fixation, Percutaneous leverage, Early exercise

## Abstract

**Background:**

Bony destructive injury of the calcaneus (BDIC) represents one of the most severe comminuted fractures of the calcaneus in which soft tissue coverage remains intact. The features of this injury include a collapsed articular surface, significant widening, severe loss of height and an unrecognisable outline of the calcaneus. This study aims to present the long-term outcomes of BDIC treated in a minimally invasive fashion followed by supervised early exercise.

**Methods:**

Twelve patients with unilateral BDICs were treated at our institution. The main surgical procedures included percutaneous traction and leverage reduction and internal compression fixation with anatomic plates and compression bolts. Early functional exercise was encouraged to mould the subtalar joint. The height, length and width of the calcaneus; Böhler’s and Gissane’s angles; reduction of the articular surfaces; and functional recovery of the affected feet were assessed.

**Results:**

The height, length and width of the calcaneus were substantially restored. The mean Böhler’s and Gissane’s angles of the affected calcaneus were 24.5 and 122.8 degrees, respectively. Five patients regained anatomical or nearly anatomical reduction of their posterior facets. Residual articular displacement of more than 3 mm was noted in three patients. Patients were followed for a mean of 93.9 months. The mean American Orthopaedic Foot and Ankle Society score was 83.8. Nine patients showed excellent or good results. Radiographic evidence of post-traumatic subtalar arthritis was observed in four cases. However, no subtalar arthrodesis was required.

**Conclusions:**

BDICs can be treated effectively with percutaneous reduction and internal compression fixation followed by early active exercise. This protocol resulted in satisfactory radiological and functional outcomes.

## Background

Highly comminuted calcaneal fractures can lead to devastating long-term disability [1,2], which is often life-altering for patients [3]. Highly comminuted calcaneal fractures are complex injuries and remain a great challenge for orthopaedic surgeons. It is often difficult to fixate these injuries, and special technical skills are required to secure the reduction and stability of the fragments. Both conservative and operative treatments have been attempted to manage such injuries. The reasons for conservative management include the complex anatomy, soft tissue problems and complications related to open reduction and internal fixation (ORIF) [4-7]. However, complications may also result from non-operative treatment, including incapacitating hindfoot malalignment (such as shortening, widening and varus angulation), subtalar arthrosis, peroneal tendon impingement, tibiotalar or fibulocalcaneal impingements and the technical difficulties of salvage surgery [3,8-11]. The widened heel can also be a cosmetic problem for women [12]. These reported complications support the selection of ORIF for highly comminuted intra-articular fractures of the calcaneus. Speck and Klaue treated 36 comminuted calcaneal fractures operatively and reported satisfactory clinical and radiological outcomes [13]. Their results suggest that anatomical reconstruction of calcaneal fractures is indicated even in highly comminuted fractures. However, several recent meta-analyses and prospective comparative trials have demonstrated that the evidence to support ORIF is weak compared with non-operative treatment [14-16]. In addition, soft tissue complications arising from ORIF still adversely affect the functional outcome, even when anatomical restoration of the subtalar congruency is achieved, which further limits the application of ORIF.

Many authors have developed minimally invasive or percutaneous techniques for the reduction and fixation of calcaneal fractures hoping to prevent the well-documented soft tissue complications related to ORIF techniques [17-20]. Although surgical techniques continue to evolve, the optimal treatment for highly comminuted fractures of the calcaneus continues to elude investigators. In efforts to manage these complex injuries with minimal soft tissue complications, we developed a minimally invasive technique with an emphasis on early joint exercise. The purpose of this study is to describe the features of highly comminuted calcaneal fractures and to report outcomes of such injuries treated with the proposed protocol.

## Methods

**Figure 1:**
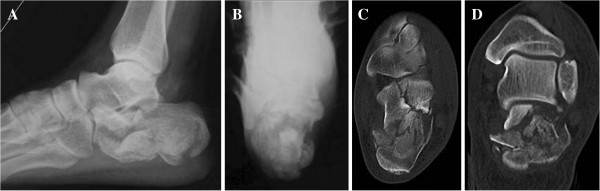
**A 53-year-old male patient suffered a left bony destructive injury of the calcaneus due to a fall from a height.** Preoperative radiographs (**A**, lateral view; **B**, axial view) and computed tomography (CT) scans (**C** and **D**, the sagittal images) demonstrate significant widening and shortening, severe loss of height of the calcaneus and collapsed articular surfaces.

From April 1, 2004, to October 31, 2006, 187 patients (215 feet) with displaced intra-articular calcaneal fractures were treated at our institute. The study was reviewed and approved by the Institutional Review Board of the Third Hospital of Hebei Medical University. Signed informed consent was obtained from each patient. The inclusion criteria were as follows: patients were adults (aged 18 years or over) with unilateral calcaneal fractures; evaluation was performed using radiography and CT scans; fractures were highly comminuted and intra-articular (Sanders type IV fractures) featuring a collapsed articular surface, more than five fracture fragments and the largest fragment diameter of less than 2.5 cm; significant shortening and widening (>25% of the normal width); more than 10% loss of height; and an unrecognisable outline of the calcaneus but with intact soft tissue coverage (Figure 1). To demonstrate the injury severity and to reflect the potentially devastating prognosis of the most serious part of Sanders type IV fractures of the calcaneus, we term such comminuted fractures bony destructive injuries of the calcaneus (BDICs). The exclusion criteria were as follows: open fractures; fractures treated using conventional plates and screws; polytraumatic injuries of the lower extremities at the time of hospital admission; and severe medical ailments.

Twelve patients with unilateral BDIC were identified. These patients included 10 males and 2 females with an average age of 39 years (range, 24–56 years); from these patients, the study involved 5 left feet and 7 right feet. Eleven patients were injured due to fall from a height, and one was involved in a traffic accident. According to the Sanders classification system [21], the BDICs were all type IV fractures. The patients underwent operative therapy at a mean of 7.6 days after injury (range, 6 to 11 days), when the swelling had subsided and positive wrinkles had appeared on the hind feet. The main surgical procedures included percutaneous traction and leverage reduction and internal compression fixation with anatomic plates and compression bolts (Shandong Wego Orthopedic Device Co., LTD). The aim was the restoration of height, length and width of the calcaneus; correction of Böhler’s angle; and anatomical or nearly anatomical reconstruction of the articular surfaces.

### Surgical technique

**Figure 2:**
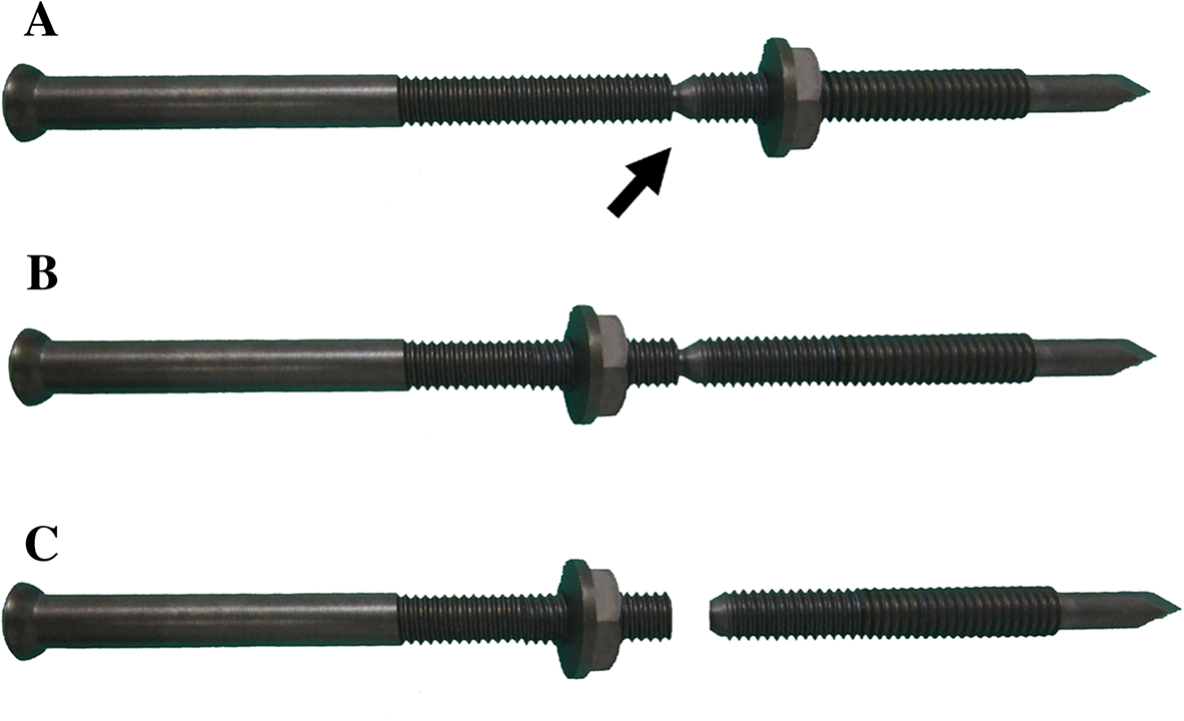
**The instruction of the compression bolts.** The compression bolts consist of nuts and screws with a constricted area (**A**; arrow). The nuts are used to fasten the screws **(B)**. The part of the screw distal to the constricted area is broken off **(C)**.

The surgical technique has been described in detail in our previous studies [22,23]. Under either epidural or spinal anaesthesia, the patients were placed in a lateral decubitus position. A C-arm fluoroscopic unit was employed to guide and assess the reduction and fixation of BDICs. During the operation, a 3.5 mm Steinmann pin was inserted into the calcaneus at the level of the calcaneal tuberosity. The pin was distracted posteroinferiorly along the long axis of the calcaneus to restore the length. Another one or two Steinmann pins were then introduced through the superoposterior portion of the calcaneus into the fragments for percutaneous leverage. Repeated leverage was recommended for a good reduction of the posterior articular surface and restoration of calcaneal height. Stress was applied to both walls of the calcaneus with clasped hands to reduce the width and height. If satisfactory reduction was achieved, the pins were advanced into the anterior part of the calcaneus for provisional fixation. A 3.5 cm longitudinal incision midway between the fibula and the Achilles tendon was made on the posterior aspect of the lateral hindfoot. An anatomical plate of the appropriate size (56.4, 60.4 or 64.4 mm in length) was selected and inserted into a subcutaneous tunnel created with a periosteal elevator. Two compression bolts (30–60 mm in length and 4 mm in diameter) were inserted into the posterior part of the hindfoot in a lateral-medial direction, and the third bolt was inserted directly into the sustentaculum tali. The patient was then placed in a supine position. Small incisions on the medial wall were made to expose the screws, and particular care was taken to avoid damaging the neurovascular bundles and tendons. The nuts (2.5 or 3.5 mm in thickness and 4 mm in inner diameter) were tightened on the screws, which were capable of generating considerable compressive force to restore the width of calcaneus to the utmost degree. Because the volume of the calcaneus is relatively constant, reducing the width can subsequently restore the height of the calcaneus (see Additional file 1). The compression bolt was broken off at the constricted area (Figure 2). Additional cancellous screws were sometimes used to secure the multi-fragmented fractures.

### Postoperative management

Postoperative radiographs and CT scans were taken to assess the quality of reduction and fixation. Supervised functional exercise was conducted to mould the subtalar joint as soon as pain was tolerable. Toe, ankle and subtalar motion was permitted on the first postoperative day. The patients began to roll the soles of their foot on a water bottle on the third postoperative day. Partial weight bearing was started at four weeks. The weight applied to the injured foot was gradually increased within the limits of patient discomfort, and the patients generally attained full weight-bearing status at 6 to 12 weeks postoperatively. Postoperative complications were recorded. Patients were followed up at one to six month intervals. Radiographs and CT scans were taken to assess the reduction and union at one, two, three, and six months and at one year, and then at six month intervals thereafter. Functional evaluation was performed according to the American Orthopaedic Foot and Ankle Society (AOFAS) scores [24]. To minimise observer bias, the questionnaires and physical examinations were all performed by one author.

### Radiological measurement

The width and length of the calcaneus were measured preoperatively and postoperatively. A 10 cm calibrator (Beijing Granville Technologies, Beijing, China) was placed close to and parallel to the sole of the foot as a measurement calibration standard when the axial and lateral radiographs of the calcaneus were taken. The images were transmitted to the PACS workstation. The width of the calcaneus was measured on the axial images with the use of the software SIENET MagicView 300 (Siemens, Erlangen, Germany), and the length of the calcaneus was measured on the lateral images.

### Statistical analysis

The data were analyzed with the use of SPSS 13.0 for Windows (SPSS Inc., Chicago, IL, USA). The one sample Kolmogorov-Smirnov test was applied to analyze the continuous variables. Continuous variables with normal distributions were expressed as the mean ± standard deviation (SD), and analyzed using the two sample *t* test. Continuous variables with non-normal distributions were recorded as the median and interquartile values, and analyzed using the Mann–Whitney *U* test. A *P* < 0.05 was considered statistically significant.

## Results

**Figure 3:**
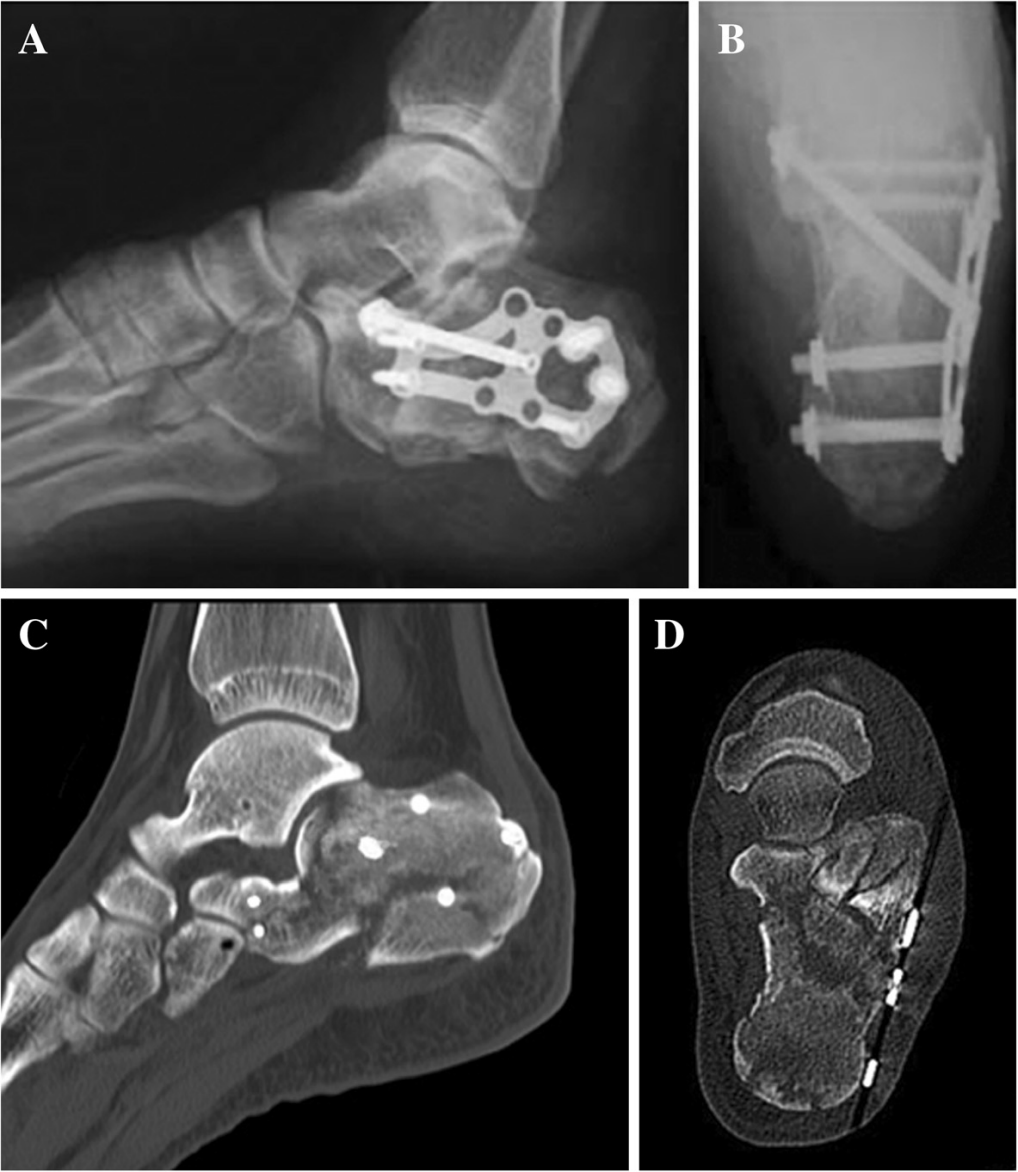
Postoperative radiographs (A, lateral view; B, axial view) and CT images (C, the sagittal image; D, the coronal image) of the same patient show that the gross anatomy of the calcaneus was restored and that the subtalar joint was significantly reduced.

The mean operative time, radiation exposure, and intraoperative blood loss plus postoperative drainage were 78 minutes (range, 56 to 93 minutes), 9.2 s (range, 6 to 15 s) and 122 mL (range, 95 to 148 mL), respectively. There were no intraoperative iatrogenic neurovascular injuries. Postoperative radiographs and CT scans demonstrated that gross anatomy of the calcaneus was restored in all patients (Figure 3). The postoperative Böhler’s angle, height, width and length of the calcaneus were significantly different from those measured preoperatively (Table 1). However, there was no significant change in Gissane’s angle. Anatomical or nearly anatomical reduction of the posterior articular surface was achieved in five patients. The posterior articular facet step ranged from 3 to 5 mm (mean, 3.9 mm) in the other seven patients according to the immediate postoperative CT scan. One patient (8.3%) developed a superficial wound infection that required a dressing change and seven days of oral cefamezin. The wound infection eventually resolved.

**Table 1:** The preoperative and postoperative radiographic measurement of the twelve calcaneal fractures

	**Pre-operation**	**Post-operation**	**P value**
**Böhler angle (°)**	19.6 ± 1.9	24.5 ± 3.7	0.001
**Gissane angle (°)**	123.6 ± 24.9	122.8 ± 11.5	0.917
**Talocalcaneal height (mm)**	29.3 ± 3.6	40.4 ± 5.1	<0.001
**Width of the calcaneus (mm)**	37.2 ± 3.9	30.8 ± 2.7	<0.001
**Length of the calcaneus (mm)**	63.4 ± 5.2	69.3 ± 5.5	0.014

**Figure 4:**
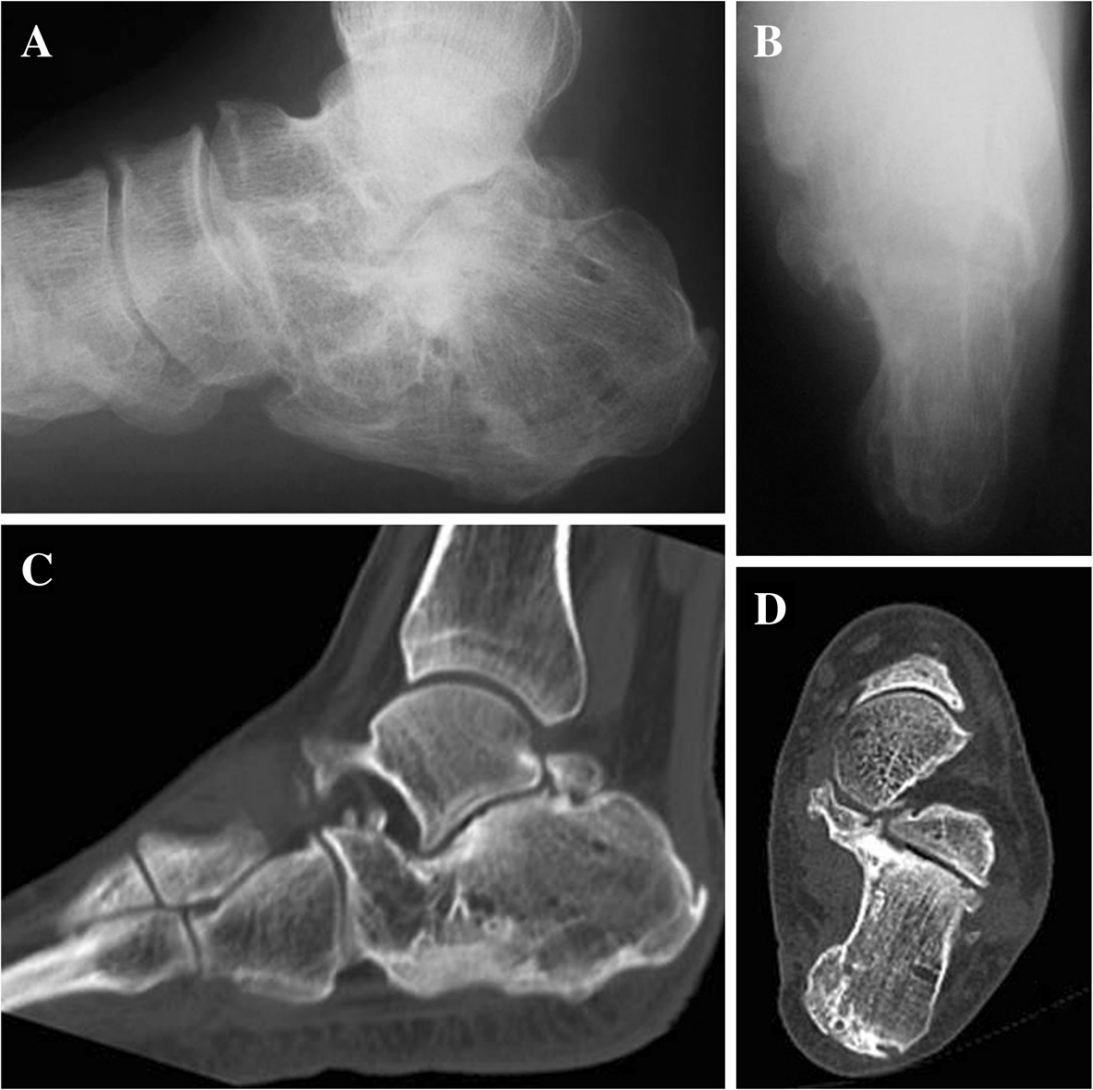
The radiographs (A, anteroposterior view; B, lateral view) and CT images (C, sagittal view; D, axial view) taken at the latest follow up demonstrate the congruent subtalar joint and degenerative changes.

Patients were followed up for an average of 93.9 ± 10.8 months (range, 79 to 115 months). Early functional exercise reduced the residual displacement of the posterior articular surface. Among the seven patients with residual articular displacement of more than 3 mm immediately after the operation, a residual displacement of greater than 3 mm remained in only three patients, and the other patients had a less than 3 mm step on the posterior facet at follow up. Radiographs and CT scans taken at the final review showed that four patients (33%) developed degenerative changes in their congruent subtalar joints (Figure 4). Among these four patients, two complained of mild pain after walking for long distances and had mild restriction when going up and down hills; one patient had moderate pain after walking two kilometres and complained of moderate restriction when he walked up or down an incline; one patient can walk normally without any pain (see Additional file 2, with signed consent obtained from this patient). Nine patients had no restriction during walking, either on flat terrain or on an incline. No patients required subtalar arthrodesis at the final follow up. The mean AOFAS score was 83.8 ± 8.0 (range, 69 to 94), and the results were graded as excellent in 4 cases, good in 5 cases, fair in 2 cases and poor in 1 case. Nine patients returned to their previous occupations within nine months after the operation. Three patients had changed to work requiring less heavy labour.

## Discussion

BDICs are highly comminuted calcaneal fractures that constitute the most severe portion of Sanders type IV calcaneal fractures. This type of injury features a significant loss of height, shortening and widening of the calcaneus, and the collapse of the articular surfaces, especially the posterior articular surface. We proposed a management protocol for such injuries, including percutaneous traction and leverage, internal compression fixation with clasped hands and an anatomical plate-compression bolt system followed by early functional exercise and weight bearing. Excellent or good functional recovery was achieved in most patients.

Highly comminuted intra-articular fractures of the calcaneus are extremely difficult to treat, and many authors have reported particularly poor prognosis [25,26]. For all practical purposes, ORIF remains the treatment of choice for such injuries at many orthopaedic trauma centres [27]. The majority of orthopaedic surgeons emphasise anatomical reduction of the articular surfaces of the subtalar joint, especially the posterior articular surface. However, simply restoring the subtalar joint surfaces does not guarantee a symptom-free foot [28]. The role of the subtalar joint as a major source of disability may have been overestimated [4]. In addition to the collapse of the posterior articular facet, the major deformities of BDICs include a significant loss of height and severe shortening and widening of the calcaneus. These deformities can lead to late sequelae. Anterior impingement may result from the loss of calcaneal height. Compression of the peroneal tendon sheath by widening the calcaneus can cause severe lateral hindfoot pain after fracture union [29]. In addition, the soft tissues also contribute significantly to residual symptoms [4]. Therefore, the reduction of the posterior articular surface is not the unique keystone to a good outcome; restoration of the calcaneal height, width and length, the mechanical axis of the hindfoot and soft tissue balance are also important prognostic factors [30-32].

In the current study, BDICs were treated with a minimally invasive surgical technique, which consists of percutaneous leverage, internal compression fixation with anatomical plates and compression bolts through small lateral incisions. Wang et al. [33] conducted a biomechanical study to compare the stability obtained using the anatomical plate and compression bolts versus that of a conventional plate and cancellous screws in the fixation of intra-articular calcaneal fractures. During 20–200 N cyclic axial loading, the anatomical plate and compression bolts showed significantly lower irreversible deformation and a higher level of fixation failure than those of the conventional plate and cancellous screws. This minimally invasive technique was applied to treat displaced intra-articular calcaneal fractures, which was demonstrated to be an effective alternative treatment, offering the combination of fewer soft tissue complications and good reduction [22,23]. In contrast to previous studies, the current study only recruited the most severe portion of Sanders type IV fractures without the inclusion of Sanders type II and type III fractures and the less severe type IV fractures. In addition to early exercise of the toe, ankle and subtalar joints, patients were instructed to roll the soles of their foot on a water bottle beginning on the third postoperative day. The twelve patients were followed up for an average of 93.9 months, which is significantly longer than the follow-up periods of the previous two studies.

Percutaneous tractional reduction with the use of Steinmann pins can restore the calcaneal length to a great degree [22,24,34]. Percutaneous leverage can primarily reduce the fractured articular surface and the calcaneal height. Both manual and implantary compression can further recreate the width and height of the calcaneus [22,24]. When recreation of gross calcaneal anatomy is obtained during the operation, walking ankle motion is recreated as well [27]. In the current study, gross anatomical calcaneal structures were achieved in all patients, and a normal walking gait was regained for each patient at the follow ups. Restoration of the gross anatomy of the calcaneus can simplify later subtalar fusions if necessary [11].

We did not use plaster for provisional fixation after the operation. Immobilisation in plaster adversely affects the end result [35,36]. Essex-Lopresti has stressed that exercise therapy has advantages over immobilisation [2]. Early motion is helpful to restore function [37,38]. In the proposed protocol for BDIC, early active mobilisation, including rolling the soles of the feet on a water bottle and early partial and full weight bearing, is an essential part of the treatment algorithm. Early functional exercise can mould the calcaneal articular surfaces via repeated compression of the talus, which contributes to a congruous subtalar joint and the restoration of Böhler’s angle [4]. For BDIC, percutaneous reduction and internal compression fixation cannot reduce all fractured fragments, especially the collapsed ones. The smaller fracture segments close to the articular surface, which were above the compression bolt inserted into the sustentaculum tali, cannot be reached and secured by a Kirschner wire or a screw, regardless of the internal fixator used during the operation. These small fracture segments cannot be reduced anatomically and fixed, and they may present as persistent displacement on the postoperative CT images. In the current study, seven patients had a displacement of greater than 3 mm on the posterior articular surface after initial operation. After the operation, supervised functional exercises were conducted to mould the subtalar joint and to further reduce any retained displaced fracture segments that were not fixed. At follow ups, improved fracture reduction was noted in four patients with a retained displacement of less than 3 mm. However, we did not consider it a significant secondary loss of reduction. Early active exercise was also useful in improving foot function and minimising the foot pain. In our study, walking with no or minimal pain was reported in nine patients.

Extensive damage to the soft tissues surrounding the calcaneus during the operation may cause residual symptoms [4]. Precise soft tissue management can minimise the risk of soft tissue complications. In this study, we treated BDICs in a minimally invasive fashion, which can reduce the surgical damage to adjacent soft tissues and subsequently reduce the risk of complications, such as wound breakdown and deep infection. Early active mobilisation can provide effective treatment for problematic soft tissues. In this case series, no patients had severe soft tissue complications. Only one patient sustained a superficial infection, which was cured by dressing changes and oral antibiotics.

The current study is limited by a small sample size. In addition, there is no control group. Controlled studies with a large sample size are needed to verify the efficiency of the proposed protocol for BDICs. Another limitation is the retrospective nature of the study. All operations were performed by one senior author, which not only minimised the bias of different surgical techniques among different surgeons but also contributed to the small sample size. In the future, when more surgeons are trained and instructed to apply this surgical technique, a randomized prospective cohort study containing a large patient population with BDICs will be possible. The minimally invasive surgical technique does not provide direct visualization of the articular facets, and can not guarantee acurate reduction of the posterior articular facet. However, this technique can achieve clinical outcome as good as, if not better than the traditional open techniques in treating displaced intra-articular calcaneal fractures [23]. The relatively bulky size of the nuts may result in potential nerve injury, skin irritation or flexor hallucis longus tendeon restriction, although no such complications were reported in the current study.

## Conclusion

BDIC is the most severe and highly comminuted type of intra-articular calcaneal fracture, and it can be treated with a minimally invasive procedure featuring percutaneous traction and leverage reduction and internal compression fixation using an anatomic plate and compression bolts. The restoration of width, height and length of the calcaneus is as important as the reconstruction of the articular congruency. Early functional exercise and weight bearing can help mould the subtalar joints, which can further reduce the retained displacement of the articular surfaces and improve the outcomes with an acceptable complication rate.

## Competing interests

The authors declare that they have no competing interests.

## Authors’ contributions

YZ, YS and WC designed the study; YS, WC and SL made substantial contributions to acquire X-ray films and CT images, and measured Böhler’s angle, Gissane’s angle and residual articular displacement; TZ organised the follow ups, and QZ evaluated the functional outcomes; TZ and QZ analyzed the data; YS, WC and SL drafted and designed the manuscript; YZ had primary responsibility for final content; All authors read and approved the final manuscript.

## Pre-publication history

The pre-publication history for this paper can be accessed here:

http://www.biomedcentral.com/1471-2482/14/19/prepub

## Supplementary Material

Additional file 1 Surgical technique using anatomical plate and compression bolt. Click here for file

Additional file 2 Patient’s walking. Click here for file
